# Metagenomic insights into the diversity of carbohydrate-degrading enzymes in the yak fecal microbial community

**DOI:** 10.1186/s12866-020-01993-3

**Published:** 2020-10-10

**Authors:** Ga Gong, Saisai Zhou, Runbo Luo, Zhuoma Gesang, Sizhu Suolang

**Affiliations:** 1 Department of Animal Science, Tibet Agricultural and Animal Husbandry College, Linzhi, Tibet China; 2 Animal Epidemic Prevention and Control Center of Tibet Autonomous Region, Lasa, Tibet China

**Keywords:** Yak, Microbiome, Carbohydrate degradation, Lignocellulolytic enzymes, Plant polysaccharides, Taxonomic diversity, Metagenome-assembled genomes

## Abstract

**Background:**

Yaks are able to utilize the gastrointestinal microbiota to digest plant materials. Although the cellulolytic bacteria in the yak rumen have been reported, there is still limited information on the diversity of the major microorganisms and putative carbohydrate-metabolizing enzymes for the degradation of complex lignocellulosic biomass in its gut ecosystem.

**Results:**

Here, this study aimed to decode biomass-degrading genes and genomes in the yak fecal microbiota using deep metagenome sequencing. A comprehensive catalog comprising 4.5 million microbial genes from the yak feces were established based on metagenomic assemblies from 92 Gb sequencing data. We identified a full spectrum of genes encoding carbohydrate-active enzymes, three-quarters of which were assigned to highly diversified enzyme families involved in the breakdown of complex dietary carbohydrates, including 120 families of glycoside hydrolases, 25 families of polysaccharide lyases, and 15 families of carbohydrate esterases. Inference of taxonomic assignments to the carbohydrate-degrading genes revealed the major microbial contributors were *Bacteroidaceae*, *Ruminococcaceae*, *Rikenellaceae*, *Clostridiaceae*, and *Prevotellaceae*. Furthermore, 68 prokaryotic genomes were reconstructed and the genes encoding glycoside hydrolases involved in plant-derived polysaccharide degradation were identified in these uncultured genomes, many of which were novel species with lignocellulolytic capability.

**Conclusions:**

Our findings shed light on a great diversity of carbohydrate-degrading enzymes in the yak gut microbial community and uncultured species, which provides a useful genetic resource for future studies on the discovery of novel enzymes for industrial applications.

## Background

Domestic yaks (*Bos grunniens*) are important livestock that can provide food and livelihood for millions of people living in the Qinghai-Tibet Plateau [1]. Yaks graze on grasses, straw, and lichens, which are plant materials rich in lignocellulosic biomass, such as cellulose, hemicellulose, and starch particles [2, 3]. Digestion of complex dietary fiber composed of plant cell wall polysaccharides and resistant starch is essential for preserving numerous physiological processes and host energy metabolism. Since the mammalian genomes generally encode few enzymes linked to digestion [4], a consortium of gastrointestinal microorganisms that harbor multiple carbohydrate-metabolizing enzymes play a significant role in the breakdown of structural polysaccharides, particularly for those found in the plant cell wall (PCW) [5, 6]. The major component of PCW polysaccharides is cellulose, which is made of β-1,4-linked Glucose polymers surrounded by a hydrated matrix consisting of hemicellulose, pectin, and lignin resistant to degradation [7, 8]. Transformation of dietary carbohydrates into soluble oligosaccharides and fermentable monosaccharides for further energy production is a crucial biological process, which requires synergism of microbial carbohydrate-degrading enzyme activities, including glycoside hydrolases, pectate lyases and carbohydrate esterases [9, 10].

In the last decade, next-generation sequencing (NGS) techniques have fueled the rapid development of metagenomics, which has the potential to investigate DNA sequences and protein-coding genes of all microbial genomes, particularly for those from hard-to-culture species [6]. Brulc et al. were the first to apply metagenomic sequencing techniques for investigation of the glycoside hydrolases in the bacterial community of dairy cows [11]. Since then, microbial diversity and the profiles of carbohydrate-degrading enzymes have been extensively studied in the gastrointestinal microbiomes of many vertebrate species [6, 12]. A study of the Asian Elephant fecal microbiota indicated that the cellulase genes belonging to glycoside hydrolase families 5 and 9 are mostly derived from *Bacteroidetes* [13]. More recently, many researchers have enabled near-complete microbial genomes from deep sequencing data through the improved analytical technique, metagenomic binning. For instance, the metagenomic analysis on the camel rumen microbiota has reconstructed 65 prokaryotic genomes and further revealed the presence and absence of genes encoding glycoside hydrolases related to lignocellulosic degradation [14].

To date, several studies on the yak gastrointestinal microbial community by NGS have been reported. The cellulolytic microbiome of the yak rumen has been investigated based on 454 pyrosequencing of 223 BAC clones and total community DNA as well [2]. Recently, a comparison of fecal bacterial communities in high-altitude mammals through 16S rRNA amplicon sequencing has revealed that the gut microbial profile of yak is distant from those of Tibetan sheep and low-altitude ruminants [1]. However, the current information about the yak intestinal microorganisms and their lignocellulolytic ability is still poor. Therefore, we investigated community structure and carbohydrate-degrading genes from the yak fecal microbiota using deep metagenomic sequencing by Illumina. A reference catalog of microbial genes was first established to explore the diversity of genes encoding carbohydrate-degrading enzymes, many of which may be novel enzymes of industrial interests. We also applied metagenomic binning to explore lignocellulolytic enzymes encoded in the recovered prokaryotic genomes.

## Results

### General features of the metagenome

The metagenome sequencing experiment of five yak fecal samples produced approximately 312 million paired reads and 92 Giga base pairs (Gbps) in total (Additional file 1). After de novo assembly using pooled sequence data from all samples, the resulting metagenome was composed of 1,676,522 contigs, with the average GC% content of 44.3% and the N50 value of 2153 bp. Among these contigs, the longest one was 377,952 bp. About 68% of the high-quality reads can be recruited back to the assembled contigs greater than 1000 bp, and the mean sequencing depth of these contigs was 26-fold, giving adequate coverage for the assembly of metagenomic reads. Gene calling based on the contig assemblies predicted 4,570,557 coding sequences (CDSs) with an average length of 698 bp. In this catalog of microbial genes, 44% (2,013,063 genes) possessed complete open reading frames with a mean length of 737 bp. The protein sequence similarity analysis showed that 70.9% (3,241,667) of all the CDSs were annotated by the entries in the NCBI non-redundant protein sequence (NR) database, 51.7% (2,363,314) annotated by the Clusters of Orthologous Groups (COG) database, 46.8% (2,136,681) annotated by the KEGG database and 61.6% (2,815,543) annotated by the Pfam database. Besides, classification of all CDSs based on the COG functional categories indicated that 11.5% were associated with information storage and processing, 10.7% with cellular processes and signaling, 17.7% with the metabolism of various biopolymers (e.g. carbohydrates, amino acids, nucleotides, coenzymes, lipids, and inorganic ions), and 0.7% with the mobile genetic materials like transposons and prophages (Additional file 2).

### Taxonomic composition of the yak gut microbiota

**Fig. 1:**
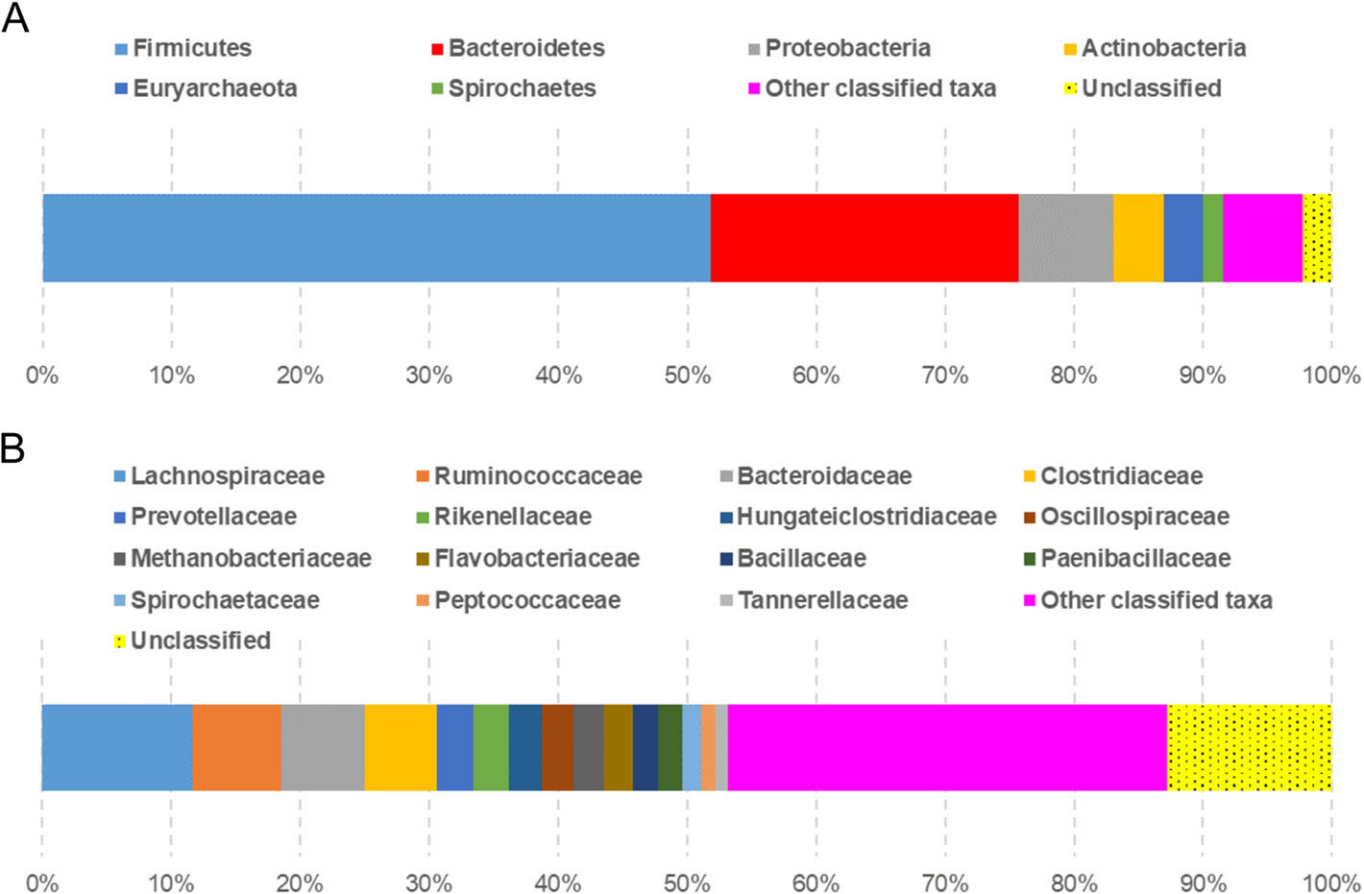
Community composition of the yak fecal microbiome. Taxonomic distribution of the microbiota based on relative abundances of metagenomic reads assigned to the phylum-level (**a**) and family-level (**b**) taxa using Kaiju [15]. Labels denote the most prevalent taxa with relative abundance ≥1%

To understand the community structure of the yak fecal microbiome, taxonomic distribution based on the pooled reads from all samples was analyzed using protein-level sequence classification. The taxonomic profile of the microbial community consisted of twenty phyla and 120 genera (≥ 0.1% abundance) (Additional file 3). *Firmicutes* and *Bacteroidetes* were the most predominant bacteria, accounting for over three quarters (75.7%) of the whole microbial community (Fig. 1a). Both phyla are also the predominant bacterial populations in the fecal microbiota of cattle [12, 16]. The other bacterial phyla with moderate abundance were *Proteobacteria* (7.3%), *Actinobacteria* (4.0%), and *Spirochaetes* (1.6%). For the archaeal domain, *Euryarchaeota* (3.0%) was the major phylum dominated in the yak fecal microbiome. At the family level, 103 families were detected and the highly abundant taxa with more than 1% abundance are displayed in Fig. 1b. It was noted that eight *Firmicutes* families were highly abundant, including *Lachnospiraceae* (11.7%), *Ruminococcaceae* (6.9%), *Clostridiaceae* (5.6%), *Hungateiclostridiaceae* (2.6%), *Oscillospiraceae* (2.5%), *Bacillaceae* (2.0%), *Paenibacillaceae* (1.8%), and *Peptococcaceae* (1.1%). A substantial diversity of the *Bacteroidetes* organisms was also found, which was well represented by five abundant families *Bacteroidaceae* (6.5%), *Prevotellaceae* (2.8%), *Rikenellaceae* (2.8%), *Flavobacteriaceae* (2.2%) and *Tannerellaceae* (1.0%). Additionally, the taxonomic profiles of individual fecal samples were also summarized in Additional file 3. As shown in Additional file 4, it seemed that the community structures of different samples were similar to each other. Based on the ANOSIM test, there was no significant difference for the microbial communities between the two study sites (*R* = 0.75, *P* = 0.10).

### Novel CAZymes in the yak gut microbiome

To explore the enzyme repertoire for the breakdown of complex polysaccharides, the genes encoding carbohydrate-active enzymes (CAZymes) present in the yak fecal microbiome were further detected using dbCAN2 [17]. It resulted in 119,926 putative CAZyme sequences assigned to 268 enzyme families, accounting for ~ 2.6% of the total genes in the catalog. To estimate the novelty of the annotated CAZymes, the protein sequences were searched against the NCBI NR database and the results were summarized in Additional file 5. A small fraction (16.2%) of all the predicted CAZymes were relatively conserved proteins that shared more than 70% identity with the best-hitting homologs. It suggested that 100,543 of the predicted carbohydrate-metabolizing enzymes may be novel, especially for 16,546 proteins that had less than 40% identity with the known proteins in the NR database.

**Fig. 2:**
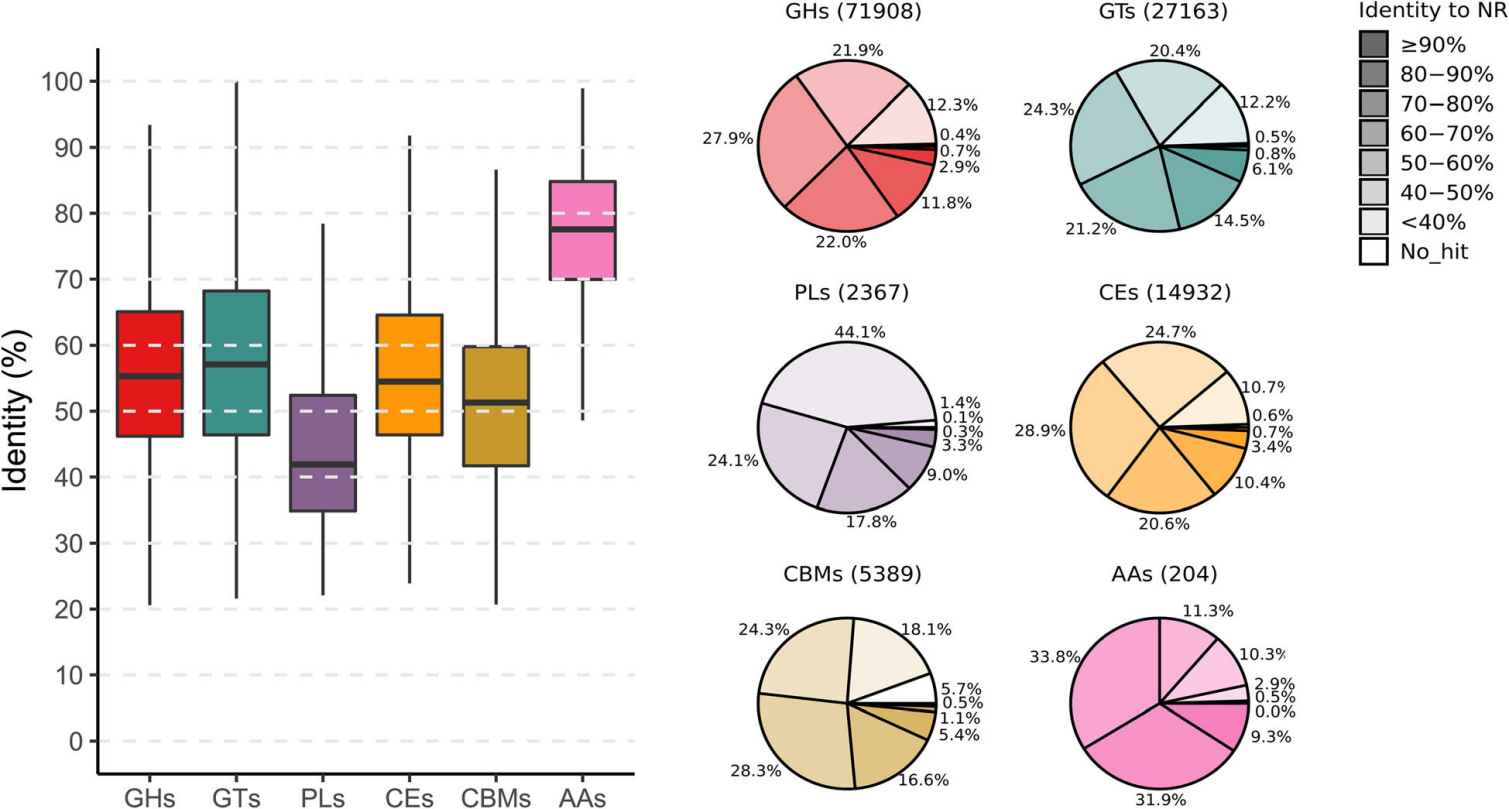
Sequence conservation of carbohydrate-active enzymes encoded in the yak fecal metagenome. The distribution of the percentage sequence identity between the annotated CAZymes and the best hits in the NCBI NR protein database is displayed by the box-plot (**a**) and pie-chart (**b**), respectively. The proteins allocated to six functional classes (i.e. GHs, GTs, PLs, CEs, CMBs, AAs) of CAZymes are separately shown. The percentage identity intervals are illustrated by the gradient of color

All the detected genes coding for CAZymes were further assigned into six functional classes: 71,908 glycoside hydrolases (GHs), 27,163 glycosyltransferases (GTs), 2367 polysaccharide lyases (PLs), 14,932 carbohydrate esterases (CEs), 5389 carbohydrate-binding modules (CBMs), and 204 auxiliary activity enzymes (AAs), respectively. The sequence conservation of these CAZymes was also evaluated through binning their identity percentages with the best matches in the NCBI NR database and the overall identity distribution is displayed in Fig. 2. It was apparent that the GHs were the most abundant, representing the majority (60.0%) of all the CAZyme genes. On the contrary, the AAs (0.2%) were very scanty in the community, and they were relatively conserved compared to the publicly available sequences, with a mean identity of 76%. Notably, the low abundant PLs (2.0%) exhibited the highest genetic divergence with a mean identity of 44%. Besides, the identity percentages for the other four classes were 58% (GTs), 56% (GHs), 56% (CEs), and 51% (CBMs), respectively.

### Diversity of carbohydrate-degrading enzymes in the microbiome

GHs (EC 3.2.1.-) are prominent enzymes for hydrolyzing the glycosidic bonds of carbohydrate substrates such as plant cell walls, starch particles, and mucin [4, 10]. Currently, sequence similarity-based family classification of CAZymes has produced 167 GH families (http://www.cazy.org/), many of which group together enzymes with different substrate activities [5]. In the yak fecal microbiome, a total of 71,908 GHs were allocated to 120 CAZy families (Additional file 6). The top 11 abundant families (i.e. GH13, GH2, GH3, GH78, GH43, GH20, GH109, GH29, GH25, GH77, and GH36) possessed 36,283 genes, accounting for about half of the total number of the GH-related sequences. GH13, which is a main α-amylase family that hydrolyzes the internal α-1, 4-glucosidic linkages of starch-related carbohydrates [20], was the largest family with a relative abundance of 8.8%. In addition, the sets of genes encoding four categories of lignocellulolytic enzymes (i.e. cellulases, endo-hemicellulases, debranching enzymes, and oligosaccharide degrading enzymes) from 26 GH families were identified in the fecal microbiome of yak (Table 1). The oligosaccharide degrading enzymes (27.0%) were the most dominating, followed by debranching enzymes (6.1%), endo-hemicellulases (4.1%), and cellulases (2.6%). The cellulases responsible for hydrolyzing β-1,4 linkages in cellulose chains were mainly represented by the genes belonging to the GH5 (cellulases) and GH9 (endoglucanase). The genes coding for endo-hemicellulases were distributed in six families GH8, GH10, GH11, GH26, GH28, and GH53. Of these, GH28 (polygalacturonase), GH10 (endo-1,4-β-xylanase), GH53 (endo-β-1,4-galactanase), and GH26 (xyloglucanase) were more abundant, accounting for nearly 97% of total endo-hemicellulases. Besides, the genes encoding debranching enzymes were mostly assigned to the families GH78 (α-L-rhamnosidase) and GH51 (α-L-arabinofuranosidase), with 3585 and 726 genes, respectively. High numbers of genes encoding different oligosaccharide degrading enzymes, e.g. β-galactosidase, β-glucosidase, β-xylosidase, α-L-fucosidase, and α-Mannosidase, were found in the families GH1, GH2, GH3, GH29, GH35, GH38, GH39, GH42, GH43, and GH94. Of these, GH2, GH3, and GH43 were the predominant enzyme families, with a relative abundance of 7.5, 5.9, and 4.9%, respectively.

**Table 1:** Comparison of the genes encoding GHs in yak fecal microbiome with five other herbivorous microbiomes

GH family	Major activity	Yak feces	Cow feces [5]	Elephant feces [13]	Termite gut [18]	Cow rumen [19]	Camel rumen [14]
Cellulases							
GH5	Cellulase	2.12	2.63	4.43	7.36	4.76	4.44
GH9	Endoglucanase	0.45	0.77	1.25	1.63	2.23	1.97
GH44	Endoglucanase	0.01	0.00	0.04	0.54	0.08	0.05
GH45	Endoglucanase	0.00	0.00	0.08	1.09	0.41	0.13
GH48	Cellobiohydrolase	0.00	0.00	0.02	0.00	0.03	0.00
Sub-total (%)		2.59	3.40	5.82	10.63	7.52	6.59
Endo-hemicellulases							
GH8	Endo-1,4-β-Xylanase	0.10	0.26	0.59	2.18	1.11	0.66
GH10	Endo-1,4-β-Xylanase	0.84	1.06	2.03	5.45	2.28	2.46
GH11	Xylanase	0.04	0.00	0.16	1.63	0.50	0.23
GH26	Xyloglucanase	0.37	1.04	0.90	2.18	1.08	1.43
GH28	Polygalacturonase	2.21	1.01	2.43	1.36	1.54	3.11
GH53	Endo-β-1,4-Galactanase	0.57	0.82	0.84	0.82	1.05	1.52
Sub-total (%)		4.14	4.19	6.95	13.62	7.56	9.41
Debranching enzymes							
GH51	α-L-arabinofuranosidase	1.01	1.13	1.93	0.82	1.85	2.48
GH54	α-L-arabinofuranosidase	0.02	0.04	0.10	0.00	0.16	0.08
GH67	α-Glucuronidase	0.08	0.22	0.33	1.36	0.55	0.59
GH78	α-L-rhamnosidase	4.99	2.85	3.63	0.00	3.21	2.34
Sub-total (%)		6.09	4.25	5.99	2.18	5.77	5.48
Oligosaccharide degrading enzymes							
GH1	β-glucosidase	0.87	0.24	0.51	1.63	0.36	0.26
GH2	β-galactosidase	7.49	6.84	7.00	2.45	6.73	6.96
GH3	β-glucosidase	5.88	5.27	6.22	6.54	8.04	7.39
GH29	α-L-fucosidase	2.77	2.84	2.93	0.27	2.00	1.62
GH35	β-galactosidase	0.64	0.51	0.81	0.27	0.32	1.15
GH38	α-Mannosidase	1.74	0.35	0.80	1.36	0.64	0.26
GH39	β-xylosidase	1.54	0.27	1.16	1.91	0.93	0.44
GH42	β-galactosidase	0.57	0.27	0.34	2.18	0.33	0.19
GH43	β-xylosidase	4.91	6.09	7.23	4.63	6.26	10.56
GH52	β-xylosidase	0.01	0.00	0.01	0.27	0.00	0.00
GH94	Cellobiose phosphorylase	0.60	0.57	1.01	8.45	1.32	0.82
Sub-total (%)		27.03	23.26	28.02	29.97	26.95	29.64
No. of all genes encoding GHs		71,908	5465	16,852	367	9897	15,959
Metagenome size		3.51 Gb	0.31Gb	0.93 Gb	0.018Gb	0.79Gb	0.66 Gb
GHs/Mbp		20.5	17.6	18.1	20.4	12.5	24.2

The table shows the statistics for each microbiome as follows: the percentages of genes belonging to distinct GH families involved in lignocellulose degradation, the number of all genes encoding GHs, the number of total bases in the assembled contigs, and the density of the GH genes in the metagenome assemblies of individual herbivores

Furthermore, the density of the GH genes in the yak fecal microbiome was 20.5 GHs per million base pairs of the assembled contigs. The comparison of GH frequencies with those present in the other herbivore microbiomes implicated that the density of GHs in yak gut was comparable to that (20.4 GHs/Mbp) of termite gut but relatively higher than that in the elephant gut (18.1), cow gut (17.6) and rumen (12.5) (Table 1). The highest density of GHs was found in the camel rumen (24.2). Meanwhile, the number of different GH families predicted in the above herbivore metagenomes was 118 in elephant gut, 112 in camel rumen, 111 in cow rumen, 97 in cow gut, and 57 in termite gut, respectively. However, the analysis also found that the GH genes were significantly overrepresented in 18 families (i.e. GH1, GH4, GH20, GH24, GH29, GH33, GH37, GH38, GH39, GH78, GH79, GH84, GH85, GH109, GH110, GH123, GH141, and GH163) in the fecal microbiome of yak comparing to the rumen microbiome of cow and camel (*p*-value < 0.01; Additional file 6). The evidence for high-density GHs and diversified enzyme families present in the fecal microbiome of yak revealed that its intestinal microbiota likely had strong potential to breakdown various plant-derived polysaccharides in vivo.

PLs (EC 4.2.2.-) are the enzymes that cleave uronic acid-containing polysaccharides using an β-elimination mechanism [21]. These enzymes can target PCW polysaccharides (e.g. pectin and pectate) and/or animal glycans (e.g. chondroitin, heparin, and hyaluronan) [4]. Here we identified 2367 genes encoding PLs fell into 25 families. Among these PLs, the common enzymatic activities related to degradation of animal glycan were hyaluronate lyase, gellan lyase, chondroitin lyase, and heparin lyase [22], which were represented by the prominent families PL35, PL33, PL12, PL8 and PL21 in the yak fecal microbiota. PL35 (447 genes) and PL33 (401 genes) were the most abundant families, both of which were significantly overrepresented in the fecal microbiota of the yak when compared to the rumen microbiota of cow and camel (Additional file 6). The lower frequencies of the PL genes encoding pectin lyase, pectate lyase and rhamnogalacturonan endolyase were found in the families PL1, PL11, and PL9, which have been reported to be involved in the breakdown of pectin and pectate that are common ingredients of PCW polysaccharides [7].

CEs are a class of esterases that catalyze the *O*-de- or *N*-deacylation of substituted saccharides and cooperate with GHs to break down PCW polysaccharides [8]. According to the CAZy database, CEs have been segregated into 17 CAZy families. The esterases in the families CE1–7 and CE16 have been supposed to deacetylating enzymes for the breakdown of acetylated plant hemicellulose [23]. In the present study, the set of the predicted CEs belonged to 15 families. Among these, CE1 (3399 genes) and CE4 (3048 genes) were the most abundant families, both representing the enzymic activity to degrade acetyl xylan. In addition, moderate abundances were also observed in the families CE2, CE3, CE6, CE7, and CE12 associated with degradation of acetylated plant hemicellulose (Additional file 6).

### Major carbohydrate-degrading genes originated from *Firmicutes* and *Bacteroidetes.*

**Fig. 3:**
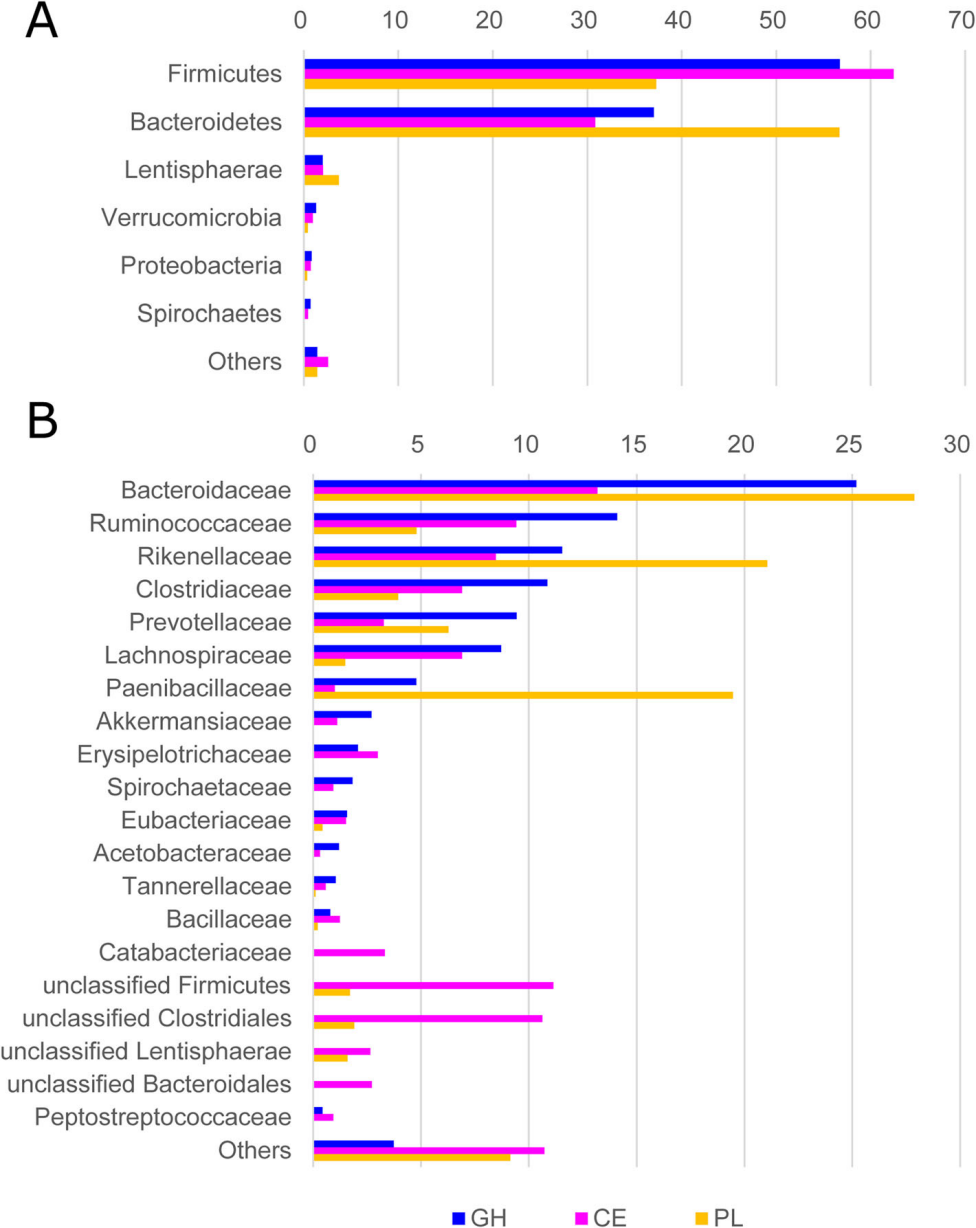
Comparison of taxonomic assignment to the genes encoding CAZymes with a role in polysaccharide degradation. Phylum- (**a**) and family-level (B) taxonomic assignments are shown for the genes coding for three CAZyme classes GHs, CEs, and PLs, respectively. The abscissa denotes the percentage of genes affiliated to the individual taxa. The ordinate denotes the detected taxa with relative abundance ≥0.5% in at least one class

To find out the major microbial populations contributing to the digestion of complex carbohydrates, taxonomic profiles of the genes encoding carbohydrate-degrading enzymes represented by GHs, CEs, and PLs, respectively, were determined by the LCA algorithm using MEGAN [24]. As shown in Fig. 3a, the majority (> 90%) of all carbohydrate-degrading enzymes were mainly derived from the microbes affiliated to *Firmicutes* and *Bacteroidetes*. Moreover, the largest cohort of microbes contributing to the gene repertoire of GHs (56.7%) and CEs (62.7%) are *Firmicutes*. By contrast, *Bacteroidetes* was the most dominant among the putative microbial producers for PLs (56.7%). A further view at the lower taxonomic level revealed that the microbial species belonging to the families *Bacteroidaceae*, *Ruminococcaceae*, *Rikenellaceae*, *Clostridiaceae*, and *Prevotellaceae* were frequently observed in all three classes of CAZymes (Fig. 3b). The proportions of PLs originated from *Rikenellaceae* (21.1%) and *Paenibacillaceae* (19.5%) were much higher than those of GHs and CEs. It was apparent that the members of *Catabacteriaceae* carried the genes encoding CEs (3.3%) alone. Additionally, the carbohydrate-degrading genes excluding the PLs were detected in the families *Akkermansiaceae*, *Erysipelotrichaecea*, *Spirochaetaceae*, and *Acetobaceraceae*. Notably, a substantial number of CEs (27.1%) were found in the cohort of unclassified bacterial species from the taxonomic clades *Firmicutes*, *Clostridiales*, *Lentisphaerae*, and *Bacteroidales* in which less abundant PLs and none of GHs were observed. The high proportion of the unclassified taxa indicated that many microbes with special metabolic potential were undiscovered in the gut community of the yak.

### Recovery of the uncultured genomes and their lignocellulolytic potential

In the present study, 68 metagenome-assembled genomes (MAGs) with completeness ≥80% and contamination ≤10% were recovered to further explore genome biology of individual lignocellulolytic species in the yak digestive tract. The characteristics of the genome assemblies and the predicted taxonomy were summarized in Additional file 6. The sizes of the MAGs were ranged from ~ 0.85 to ~ 3.53 Mb with an average N50 of 26,918 bp. Additionally, these MAGs harbored a varied GC% content between 22.9 to 64.1%, representing a broad range of diverse microbes (Additional file 7).

**Fig. 4:**
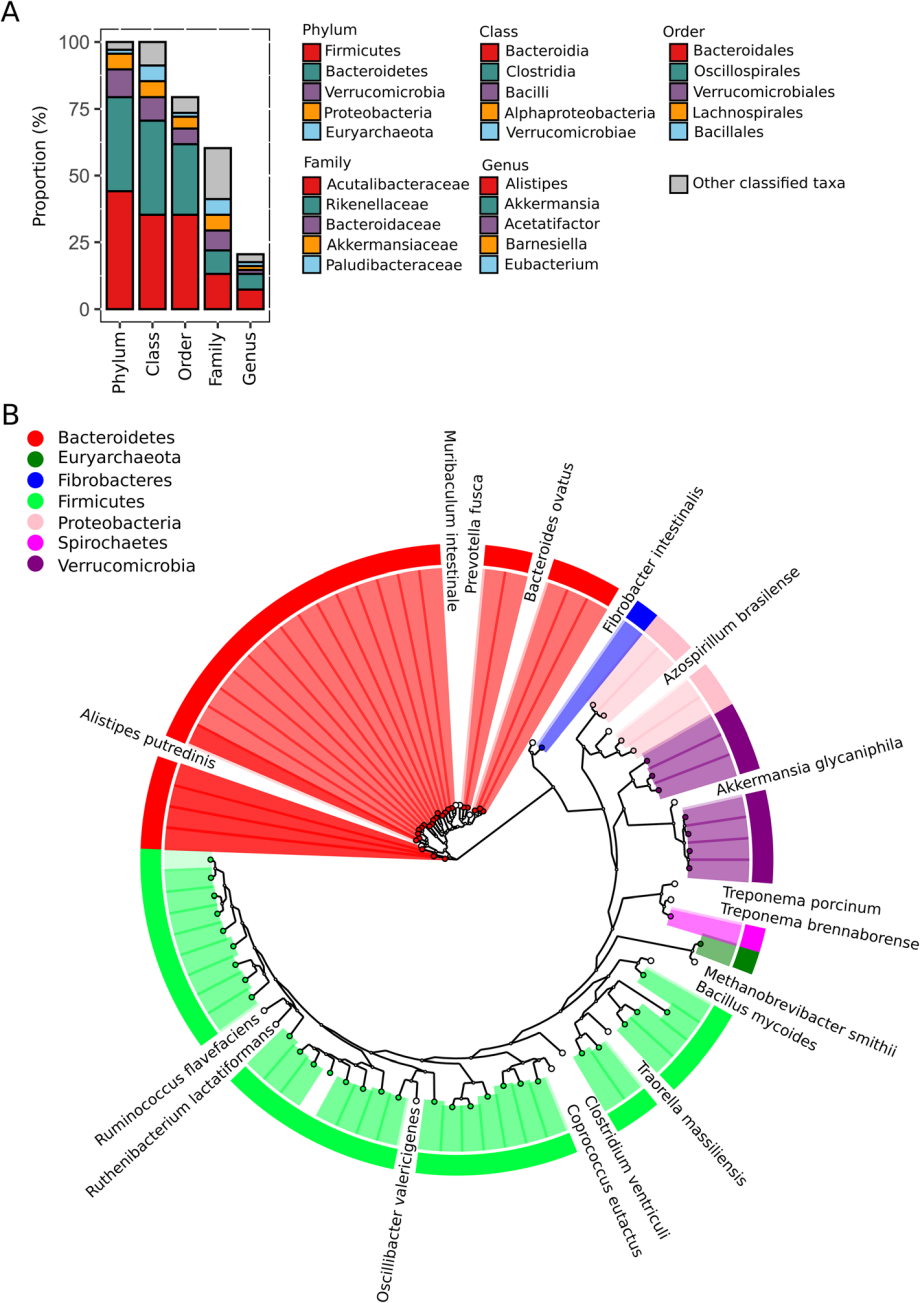
Taxonomic and phylogenetic structure of the uncultured gut prokaryotic species. **a** The stacked bar plot showing the five most prevalent taxa at the phylum, order, class, family, respectively. **b** Circular Phylogram of 68 metagenome-assembled genomes and the species representative genomes retrieved from the NCBI RefSeq database. The outermost color strips denote the phylum-level taxa of the draft genomes corresponding to the tips of the phylogenetic tree. The colored tip nodes denote the genome bins and the white nodes for the public reference genomes

The analysis of taxonomic inference for MAGs indicated that all the putative genomes were assigned to six bacterial phyla and a single archaeal phylum (Fig. 4). Among the MAG-representing microbial populations, the most frequently observed taxa were the species affiliated to *Firmicutes* (30 MAGs), followed by *Bacteroidetes* (24 MAGs). The other MAGs were taxonomically assigned to the phyla *Verrucomicrobia* (7 MAGs), *Proteobacteria* (4 MAGs), *Fibrobacteres* (1 MAG), *Spirochaetes* (1 MAG), *Euryarchaeota* (1 MAG). All MAGs of *Bacteroidetes* belonged to the order *Bacteroidales*, and the majority (24 out of 30) of *Firmicutes* MAGs were affiliated to the class *Clostridia*. As shown in Fig. 4a, the number of genomes that could be taxonomically classified was decreased sharply at the genus level. Approximately a quarter (14 out of 68) of the MAGs were classified at the genus level, and none of the genomes were assigned with the taxonomic identifiers at the species level (Additional file 7). It suggested that most of the uncultured genomes were novel species firstly discovered in the yak gut microbial community.

On the other hand, based on phylogenetic reconstruction with 400 highly conserved prokaryotic proteins, the whole-genome phylogeny for the uncultured genomes together with some public reference genomes from the closely related species is illustrated in Fig. 4b. The genetic relationships of the MAGs were consistent with their taxonomic classifications at the phylum level. For instance, a single *Euryarchaeota* MAG (#32) clustered with *Methanobrevibacter smithii* was assigned to the genus *Methanobrevibacter*, which has been identified as the dominant methanogen in the large intestine of finishing pigs [25].

**Fig. 5:**
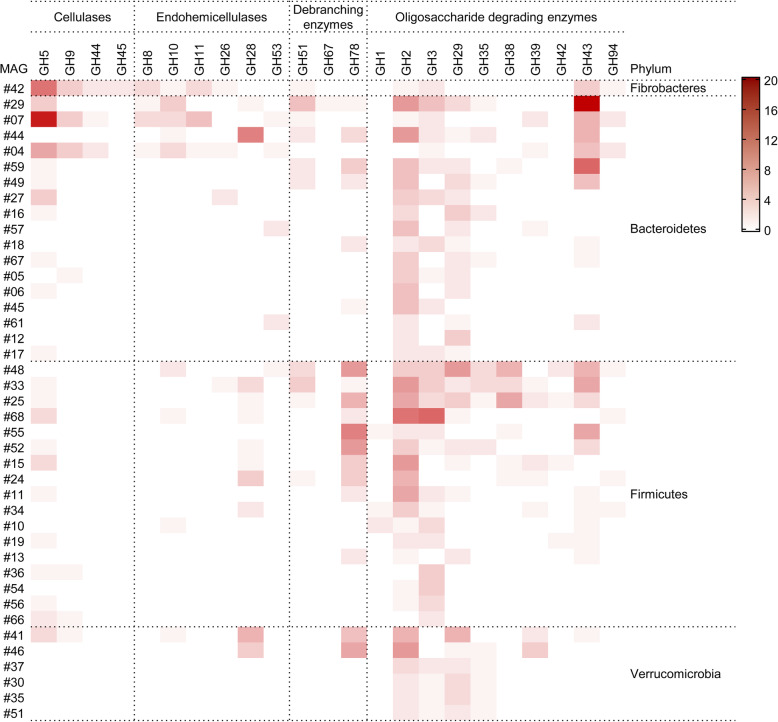
Distribution of the GH families associated with the major lignocellulolytic enzymes across the recovered genomes. The heatmap shows the frequency of the genes affiliated to individual GH families. Only the MAGs carrying at least five genes belonging to any GH are displayed herein

In terms of the genes coding for GHs in the recovered genomes, the potential of lignocellulosic degradation was evaluated. The amount and gene density of all GH-encoding genes present in the genomes belonging to different phyla is shown in Table 2. A lot of GHs were observed in the genomes of *Bacteroidetes* and *Firmicutes*, which possessed 897 and 857 genes, respectively. The highest gene density, 27.0 GHs/Mbp, was observed in the *Fibrobacteres* MAG (#42) that was assigned to the family *Fibrobacteraceae*. The relatively high density of GHs was found in the bacterial genomes from the phyla *Bacteroidetes* (19.5), *Firmicutes* (17.6), and *Verrucomicrobia* (17.8), respectively (Table 2). Conversely, the GHs are scarce in the single *Euryarchaeota* MAG, which encodes two GH genes only. Besides, the distribution of the GHs involved in the degradation of PCW polysaccharides across the MAGs is displayed in Fig. 5. The genes encoding lignocellulolytic enzymes were frequently distributed in the following families: cellulases (GH5), endo-hemicellulases (GH10 and GH28), debranching enzymes (GH51 and GH78), and oligosaccharide degrading enzymes (GH2, GH3, GH29, GH38, GH39, and GH43). It was worth noting that 15 MAGs were derived from novel bacteria species with carbohydrate-digestive capacity, each with more than 20 genes encoding lignocellulolytic enzymes. They were seven *Clostridia* MAGs (#15, #25, #33, #48, #52, #55, and #68) belonging to the *Firmicutes*, five *Bacteroidia* MAGs (#04, #07, #29, #44, and #59) belonging to the *Bacteroidetes*, and two *Kiritimatiellae* MAGs (#41 and #46) belonging to the *Verrucomicrobiota*, and the remaining MAG (#42) belonging to the *Fibrobacteres*. Two *Bacteroidetes* MAGs (#29 and #07) harbored the most abundant (hemi)cellulose-degrading genes, with 54 and 49 genes, respectively. MAG07 possessed abundant genes encoding cellulases (18 GH5, 4 GH9, and 1 GH44) and endo-hemicellulases (5 GH11, 3 GH8, 3 GH10, and 1 GH53). By contrast, more genes encoding debranching (5 GH51, 1 GH67, and 1 GH78) and oligosaccharide degrading enzymes (20 GH43, 8 GH2, 5 GH3, 3 GH29, and 1 GH35) were detected in MAG29.

**Table 2:** Statistics of genes encoding glycoside hydrolases in the MAG-representing microbial population

Phylum^a^	Genomes	No. GHs	Mean GHs per genome	GHs/Mbp^b^
Bacteroidetes (−)	24	897	37.4	19.5
Euryarchaeota (+/−)	1	2	2.0	1.3
Fibrobacteres (−)	1	53	53.0	27.0
Firmicutes (+)	30	857	28.6	17.6
Proteobacteria (−)	4	24	6.0	5.4
Spirochaetes (−)	1	15	15.0	6.5
Verrucomicrobia (−)	7	278	39.7	17.8
Total	68	2126	31.3	17.6

^a^ Gram-positive (+) and gram-negative (−) phyla are represented in parentheses

^b^ The number of genes per 1 million base pairs of the metagenome-assembled genomes

## Discussion

The diversity of ruminal microorganisms and the profile of glycoside hydrolases bearing cellulolytic capability in the yak rumen have been depicted [1, 2, 26], but our understanding on the capability of the yak gut microbiota to digest complex dietary carbohydrates has not been well described for this important livestock so far. In this report, we utilized the sequenced fecal metagenomic data to establish a reference catalog of microbial genes and further characterize the gene products of the enzyme families GHs, PLs, and CEs associated with the breakdown of complex carbohydrates in the yak gut. Meanwhile, some uncultured genomes of novel bacterial species with lignocellulolytic potential were first identified through a metagenomic binning approach.

### Diversity of carbohydrate-degrading enzymes and microbial contributors

As is well known, the ruminant gastrointestinal microbiota can produce a wide array of CAZymes involved in the utilization of lignocellulosic biomass, which is the most abundant and bio-renewable resource on earth [14, 19, 27]. In our study, a large repertoire of genes coding for carbohydrate-degrading enzymes were identified in the yak fecal microbiome. This complex gene repertoire composed of highly diversified enzyme families should provide multiple catalytic abilities to the utilization of various carbohydrate substrates, such as plant cell walls, starch, and mucin in the yak intestinal community.

Among the carbohydrate-degrading enzymes, GHs are a key class of the predominant enzymes for the utilization of the most lignocellulosic biomass in the mammalian gut microbiota [5, 13]. Consistently, the GH genes present in the yak fecal microbiome encoded the highly diversified biomass-degrading enzymes, which were allocated to 120 GH families. Of these, the genes encoding starch-degrading enzymes of the GH13 family, the representative of the amylolytic enzyme family, were found to be the most abundant in the community. It has also been suggested GH13 is the most prevalent family in the human gut microbiota [4]. Besides, among the 11 top abundant GH families mentioned above, members of the families GH2, GH3, GH29, GH36, GH43, and GH78 have been characterized by their catalytic action modes to degrade PCW polysaccharides in the human gut microbiota [4]. Four families (i.e. GH2, GH3, GH29, and GH43) were the main enzymes responsible for oligosaccharide degradation. It was noted that the yak fecal microbiome had a higher proportion of debranching enzymes when compared with those in the microbiomes of the other five herbivores (Table 1). Among the genes encoding debranching enzymes, members of GH78 mainly acting as α-L-rhamnosidases were predominant for cleaving rhamnose from polysaccharides. The high abundance of GH78 has been also found in the microbial communities of elephant feces [13] and cow rumen [19]. The other debranching enzymes, such as β-xylosidase, α-L-arabinofuranosidase, and α-glucuronidase, which play a crucial role in depolymerization of hemicellulose [28], were also identified and represented by the genes assigned to the families GH51, GH54, and GH67. For the hemicellulose-degrading enzymes, the genes belonging to the most abundant family GH28 were coding for polygalacturonases involved in pectin digestion [29].

Many carbohydrate-degrading genes are unique and/or overrepresented in the fecal microbiota of the yak, which may contribute to the utilization of specific substrates as additional energy sources. Dai et al. [2] have reported the cellulolytic microbiome of the yak rumen and described a profile of 55 GH families based on 429 Mb metagenomic sequences. In comparison to the rumen microbiome, the fecal microbiome of the yak appears to harbor a broader spectrum of GHs, with 68 extra enzyme families. Furthermore, the frequencies of 23 CAZy families involved in complex carbohydrate degradation were significantly enriched in the fecal microbiota of yak in comparison to the rumen microbiota of other ruminants (Additional file 6). Some of these enriched families can target the substrates of both plant structural polysaccharides (i.e. GH1, GH4, GH38, GH39, and GH79) and animal glycans (i.e. GH20, GH33, GH79, GH84, GH85, GH109, GH110, GH123, PL33, and PL35) [4, 30, 31].

Certain bacterial species, notably among the *Bacteroidetes*, have been known to play a key role in degrading complex non-digestible dietary polysaccharides in the mammalian intestine [10]. Five dominating bacterial families present in the yak fecal microbial community were identified as the major contributors to produce about half of polysaccharide-degrading enzymes GHs, CEs, and PLs, respectively. Of these, *Bacteroidaceae*, *Rikenellaceae*, and *Prevotellaceae*, all belonging to the *Bacteroidetes* phylum, have been considered primary saccharolytic bacteria in many ecosystems. The other two *Firmicutes* families, *Ruminococcaceae* and *Clostridiaceae*, possessed some well-studied cellulolytic organisms, which have been experimentally verified in ruminants and pigs, such as *Ruminococcus albus*, *R. flavefaciens*, *Clostridium longisporum* and *C. herbivorans* [10, 32]. The four bacteria families (*Bacteroidaceae*, *Prevotellaceae*, *Ruminococcaceae*, and *Clostridiaceae*) dominated in the yak fecal microbiome have also been detected as the main producers for CAZymes in the cattle rumen microbiome [28]. However, the *Fibrobacteres* bacteria, which are important degraders of cellulose and are often highly abundant in the bovine rumen [33, 34], were found to be lowly abundant in the yak fecal microbiome (Additional file 3). Besides, *Paenibacillaceae* within the class *Bacilli* was the third abundant population among all microbial producers contributing to the PL-encoding genes in the fecal microbiome of yak. Some plant-associated *Paenibacillaceae* strains that may convert lignocellulosic biomass to useful products have been frequently detected in the compost microbial communities [35]. For instance, genomic analysis of the *Paenibacillus* strain P1XP2, which has been recently isolated from a commercial bioreactor degrading food waste, has uncovered genes coding for the enzymes involved in the breakdown of polysaccharides [36].

### Lignocellulolytic microorganisms in the repertoire of MAGs

To associate carbohydrate-degrading enzymes with individual microbial species/strains, we characterized the binned MAGs and the genes encoding lignocellulolytic enzymes in the fecal microbiota of yak. Interestingly, the repertoire of the MAGs was mainly represented by two bacterial clusters, *Bacteroidales* from the Gram-negative phylum *Bacteroidetes* and *Clostridia* from the Gram-positive phylum *Firmicutes*. The predominance of both clusters whose members are largely anaerobic bacteria has also been found in the sets of 913 MAGs recovered from the cow rumen [37]. Meanwhile, a recent study by Comtet-Marre et al. [38] has revealed that the majority of unclassified reads from actively expressed CAZyme genes in vivo could be mapped to the draft genomes belonging to *Bacteroidales* and *Clostridiales* in the microbial community of cow rumen [19]. Consistently, most MAGs reconstructed herein were newly discovered species within both *Bacteroidales* and *Clostridia*, which should provide reference genomes for future taxonomic study. Besides, a single MAG assigned to the phylum *Fibrobacteres* exhibited the highest density of GHs, implying its capability of degrading plant fiber. The members of *Fibrobacteres* have been considered to be the primary degraders of fibrous plant material in the gut of herbivores [39]. Both MAGs (#41 and #46) that were assigned to a recently proposed class *Kiritimatiellae* under the phylum *Verrucomicrobia* encode some GHs responsible for the degradation of both plant polysaccharides (GH2, GH5, GH28, GH29, GH36, and GH39) and animal glycans (GH2, GH20, GH95, GH109, and GH123) [4, 40].

Some microbial species contributing to the breakdown of host-derived glycans were also detected in the fecal microbial community and uncultured genomes of yak. For instance, the genera *Akkermansia* and *Bifidobacterium* were identified with a relative abundance of 0.51 and 0.29%, respectively (Additional file 3). The previous studies have pointed out that *Akkermansia* spp. isolated from the mammalian intestinal and fecal samples could produce some enzymes to degrade and utilize mucin in the gastrointestinal tract [41]; while *Bifidobacterium* spp. dominated in the feces of most infants harbor the ability to utilize oligosaccharides, e.g. L-fucose, D-glucose and D-galactose in breast milk [42]. Besides, we identified four MAGs (#30, #35, #37, and #51) belonging to the same genus *Akkermansia* (Additional file 7), whose members are gram-negative and strictly anaerobic bacteria within the phylum *Verrucomicrobia* [43]. In these MAGs, except for the plant polysaccharide-degrading genes distributed in the families GH2, GH3, GH29, and GH39 (Fig. 5), other GH families associated with the mucin-degrading enzyme activities were also detected, including β-galactosidases (GH2 and GH20), neuraminidases/sialidases (GH33), fucosidases (GH29 and GH95), exo- and endo-β-N-acetylglucosaminidases (GH18 and GH84) and α-N-acetylglucosaminidases (GH89) [31]. The evidence shown herein further confirms that some bacterial populations could utilize the host mucins as an alternate energy source for nutrient acquisition in the gut ecosystem of yak.

## Conclusions

In summary, deep metagenome shotgun sequencing was adopted to comprehensively analyze the fecal microbial community of yak. A reference catalog of gut microbial genes was established for this important herbivorous animal in the Qinghai-Tibet Plateau. We characterized a gene repertoire comprising highly diversified carbohydrate-degrading enzymes. Metagenomic binning was performed to recover 68 prokaryotic genomes and further explore the putative lignocellulolytic bacteria in the gut ecosystem of yak. These findings provide a valuable genetic resource for future discovery of novel enzymes and microbial candidates not only involved in the efficient degradation of complex plant polysaccharides, but also for industrial applications, such as food processing, biofuel, and biocatalysts.

## Methods

### Sample collection and DNA preparation

Fecal samples were collected from five female domestic yaks aged between 2 and 5 years in Qinghai-Tibet Plateau. Two sample sites were chosen and more details about geographic information were listed in Additional file 1. The sample collection was carried out according to the manufacturer’s protocol of the Longseegen Stool Storage Kit (Longsee Biomedical Corporation, China). Briefly, ~ 1 g fresh feces from each animal were picked up using the stool collection tubes, suspended in 3 ml stool storage solution and stored at − 20 °C. Total community DNA was extracted by using QIAamp DNA Stool Mini Kit (Qiagen, Germany). Quality and purity of DNA were quantified using Nanodrop ND1000 (Thermo Fisher Scientific, USA) and electrophoresis in 1% agarose gel. DNA concentration was measured using a Qubit 2.0 Fluorometer (Thermo Fisher Scientific, USA).

### Metagenomic sequencing

Whole metagenome shotgun sequencing was carried out on an Illumina NovaSeq 6000 instrument at Novogene (Nanjing, China) according to the standard protocols. A library of 300–500 bp purified DNA fragments were constructed using the TruSeq DNA library kit (Illumina Inc., USA). Briefly, ~ 2 μg DNA was sheared using the Covaris instrument (Covaris, USA) followed by end-repair, adenylation, ligation with Illumina adapters, and then amplification by eight PCR cycles. The library was quantified using Qubit 2.0 and the size of inserted fragments was checked using Agilent 2100 BioAnalyzer (Agilent, USA). Then after cluster generation in cBot, the library was sequenced in a mode of 2 × 150 bp paired-end reads.

### Sequence assembly and genome binning

Raw sequencing reads were preprocessed to trim the low-quality bases and adaptor sequences by using Trimmomatic v0.39 with options LEADING:20 TRAILING:20 SLIDINGWINDOW:4:20 MINLEN:40 AVGQUAL:20 [44]. To remove host-derived DNA contamination, the clean reads aligned to the reference genome of *Bos grunniens* (domestic yak, RefSeq assembly: GCA_005887515.2) were filtered using BMTagger implemented by MetaWRAP v1.2.2 [45, 46]. After removal of host reads, the sequence data per sample were pooled for a co-assembly using Megahit v1.1.3 [47] included in MetaWRAP, with options -t 36 -m 200 -l 1000. Only contigs more than 1 kb were retained for the subsequent analyses. To estimate the quality of assembled contigs, sequencing coverage was investigated via mapping reads to the assembled contigs by BBMap v38.73.

Next, contig binning was conducted to recover individual genomes based on their tetranucleotide frequencies and differential coverages. MetaBat v2.12.1 [48] and MaxBin v2.2.6 [49] were chosen for independent binning using contigs longer than 2000 bp and clean reads of each sample. Two sets of draft bins were further consolidated into a single bin set using the bin_refinement module of the metaWRAP pipeline with options -t 72 -m 150 -c 70 -× 10. The incorrect binned contigs were detected and removed from each MAG using MAGpurify v2.1.2 with the following modules phylo-markers, clade-markers, tetra-freq, gc-content, and known-contam [50]. For the final bins, CheckM v1.0.12 was used to estimate the genome completeness and contamination according to the 43 curated phylogenetically informative marker genes provided by this package and options lineage_wf -t 36 [51]. The draft genomes with completeness ≥80% and contamination ≤10% [37] were retained for the subsequent analyses and submission to the GenBank database.

### Taxonomic annotation of microbial community

To infer taxonomic compositions of the microbial community, the metagenomic classifier Kaiju v1.7.3 [15] was employed for profiling all the reads in the community with default parameters. Nucleotide sequences of all clean reads were translated into amino acid sequences and then used for searching against the pre-formatted NCBI RefSeq protein database. The matches were then counted according to the NCBI taxonomic lineages of the hits and the percentages of the classified reads assigned to individual taxa were defined as relative abundance. The reads mapping to the sequences of viruses and phages were discarded in this study. The statistical difference for the taxonomic profiles between study sites was estimated using the function *anosim* from the R package vegan v2.5–6 [52].

### Functional annotation of microbial community

Protein-coding sequences of the co-assembled metagenomic contigs were predicted using the software Prodigal v2.6.3 with the options -p meta -m [53]. Functional annotation of these CDSs was performed by local alignment searching against the databases NCBI NR [54] and COG [55] using DIAMOND v0.9.14 [56]. Protein structural domains were detected by homology searching against PFAM v32 [57] using *hmmscan* implemented by HMMER v3.2.1 [58]. The KEGG Orthologs were detected by searching the query proteins against the KOfam database of profile Hidden Markov Models (pHMMs) [59] using HMMER/*hmmscan*.

dbCAN2 [17] was used to predict the genes encoding CAZymes based on a set of pHMMs corresponding to the enzyme families defined by the CAZy database [60]. Currently, six major classes of CAZymes are GHs, GTs, PLs, CEs, CBMs, and AAs, respectively. Among these, the GTs are involved in the biosynthesis of carbohydrates; the GHs, CEs, and PLs break down polysaccharides; the CBMs enhance the catalytic efficiency of the above four classes; the AAs are involved in lignin degradation [3, 61]. The identifiers of CAZy families were assigned to the CDSs according to the suggested criteria for the HMMER search: E-value <1e-15 and coverage > 0.35 [17]. Multiple CAZy families present in a single sequence were allowed. To infer the microbial origin of the CAZymes, DIAMOND was used to search the query protein sequences against the NR database. For each gene, the top 20 hits with an E-value of >1e-3 were retained. Then we applied the lowest common ancestor (LCA)-based algorithm implemented by the package MEGAN v6 to determine the taxonomic level of each gene [24]. The CAZymes were then compared with several publicly available metagenomic datasets, including cow feces [5], elephant feces [13], termite gut [18], cow rumen [19] and camel rumen [14] using the same computational pipeline.

### Taxonomic, phylogenetic and functional analyses of MAGs

The taxonomic assignments for the binned genomes were performed using *classify_wf* in the Genome Taxonomy Database Toolkit (GTDB-Tk) v1.2.0 with default parameters [62]. The protein-coding genes, rRNAs, tRNAs of each MAG were predicted using the integrated pipeline Prokka v1.13 with default parameters [63]. To estimate the genetic relationships among all MAGs, a maximum likelihood phylogenetic tree was built based on a concatenated protein sequence alignment using the package PhyloPhlAn v1.0 [64]. The taxonomic and phylogenetic information were then combined and visualized by GraPhlAn [65]. Genes encoding glycoside hydrolases in the individual genomes were detected using the same procedures as those encoded in the metagenome.

## Supplementary information

**Additional file 1.** Information on the sampling and sequencing of yak feces collected in this study.

**Additional file 2.** Summary of COG functional classification of the protein-coding genes in the assembled metagenome.

**Additional file 3.** Taxonomic annotation of the yak fecal microbial community. The percentages of estimated taxa from the phylum level to the genus level in the community are shown for individual samples and pooled data.

**Additional file 4.** The bar chart showing the taxonomic distribution of five metagenomes from the yak fecal microbial community. (A) Distribution at the phylum level. (B) Distribution at the family level. The taxa with relative abundance ≥1% are shown.

**Additional file 5.** Protein sequence identity between the predicted CAZymes in the yak fecal microbiome and the best matched subjects in the NCBI NR database.

**Additional file 6 **The number of genes belonging to the enzyme families of GHs, CEs, and PLs in the gastrointestinal microbial communities of yak and the other herbivores. Fisher’s exact test was used to assess significant differences in the gene count data between the microbiomes of yak and the other herbivores. Bonferroni-corrected *p*-values were labeled as following: * for *p*-value < 0.05 and ** for *p*-value < 0.01.

**Additional file 7.** Summary of genome features and the predicted taxonomic assignments to the MAGs recovered in this study.

## Data Availability

The sequences of raw short reads generated in this study have been deposited at the NCBI SRA database under the accession number PRJNA624740. Fasta files of the assembled prokaryotic genome sequences are available from the GitHub repository at https://github.com/szslbio/Yak-MAGs.

## Abbreviations

PCW: Plant cell wall
NGS: Next-generation sequencing
CDSs: Coding sequences
CAZymes: Carbohydrate-active enzymes
GHs: Glycoside hydrolases
GTs: Glycosyltransferases
PLs: Polysaccharide lyases
CEs: Carbohydrate esterases
CBMs: Carbohydrate-binding modules
AAs: Auxiliary activity enzymes
MAGs: Metagenome-assembled genomes
NCBI NR: NCBI non-redundant protein sequence database
COG: Clusters of Orthologous Groups
pHMMs: Profile Hidden Markov Models
