# Supplementary figures and images for: Metagenomic insights into the diversity of carbohydrate-degrading enzymes in the yak fecal microbial community
Source: BMC Microbiol. 2020 Oct 10;20:302. doi: 10.1186/s12866-020-01993-3 (PMC7547465; doi:10.1186/s12866-020-01993-3)

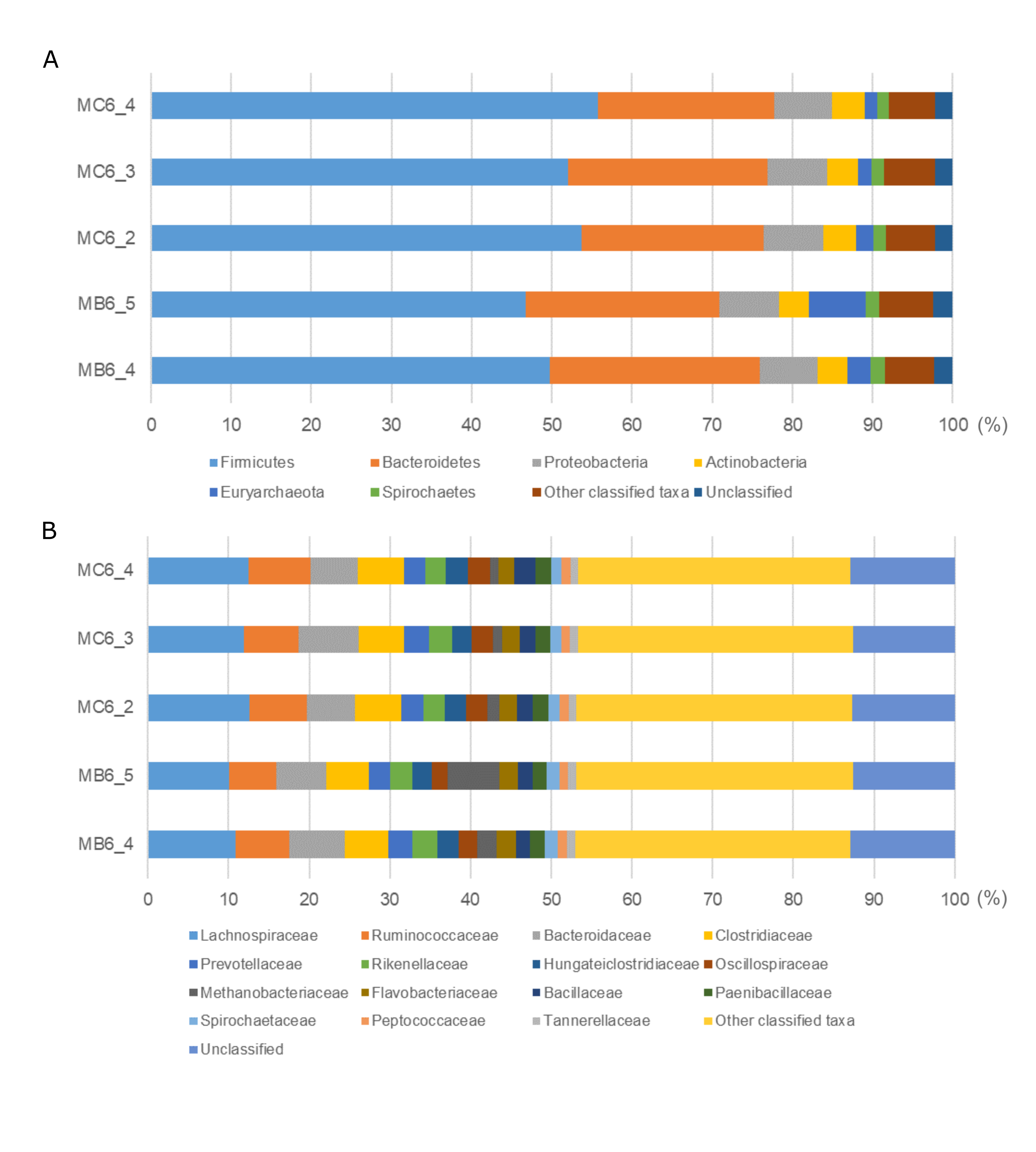

Supplement: Supplementary file 4 — Additional file 4. The bar chart showing the taxonomic distribution of five metagenomes from the yak fecal microbial community. (A) Distribution at the phylum level. (B) Distribution at the family level. The taxa with relative abundance ≥1% are shown. [file 12866_2020_1993_MOESM4_ESM.tif]
